# Consistency Analysis of Centiloid Values Across Three Commercial Software Platforms for Amyloid PET Quantification

**DOI:** 10.3390/diagnostics15131599

**Published:** 2025-06-24

**Authors:** Hyukjin Yoon, Narae Lee, Yoo Hyun Um, Woo Hee Choi

**Affiliations:** 1 Division of Nuclear Medicine, Department of Radiology, St. Vincent’s Hospital, College of Medicine, The Catholic University of Korea, 222 Banpo-daero, Seocho-gu, Seoul 06591, Republic of Korea; aksasin@catholic.ac.kr (H.Y.);; 2 Department of Psychiatry, St. Vincent’s Hospital, College of Medicine, The Catholic University of Korea, 222 Banpo-daero, Seocho-gu, Seoul 06591, Republic of Korea; cherubic712@naver.com

**Keywords:** Centiloid, Alzheimer’s disease, PET

## Abstract

**Objectives**: This study aimed to evaluate the consistency of Centiloid (CL) values calculated using three commercially available software platforms: BTXBrain (v1.1.2), MIM (v7.3.7), and SCALE PET (v2.0.1). **Methods**: A total of 239 patients who underwent amyloid PET/CT with either F-18 flutemetamol (FMM) or F-18 florbetaben (FBB) were retrospectively analyzed. CL values were calculated using BTXBrain, MIM, and SCALE PET. Linear regression, Passing–Bablok regression, and Bland–Altman analysis were performed to assess the agreement between CL values. Subgroup analyses were conducted for each radiotracer. CL values were compared according to visual interpretation status. **Results**: Strong correlations were observed between CL values derived from the three software platforms (R^2^ > 0.95). However, Passing–Bablok regression revealed significant proportional bias, with CL values from BTXBrain being lower than others, and CL values from SCALE PET being higher than others as CL values increased. Bland–Altman plots visualized the proportional bias, particularly between BTXBrain and SCALE PET. Subgroup analyses by radiotracer showed similar results. CL values in visually positive scans were significantly higher than those in visually negative scans across all platforms. **Conclusions**: The three commercial software programs demonstrated high consistency in CL quantification. However, a notable systematic bias was observed. Further evaluation of various scanner effects and CL calculation methods is warranted to improve the consistency and reproducibility of CL quantification in clinical practice.

## 1. Introduction

Alzheimer’s disease (AD) represents the predominant cause of dementia, responsible for 60–80% of cases in individuals over the age of 65 [1]. The deposition of β-amyloid (Aβ) plaques in the brain is recognized as a significant pathological hallmark of AD [2]. Positron emission tomography (PET) is a key imaging modality for visualizing amyloid burden [3]. Although visual interpretation is frequently employed in clinical environments, it has drawbacks, including variability among readers and a lack of objectivity [4]. Quantitative metrics, such as the standardized uptake value ratio (SUVr), are increasingly employed to provide an objective and reproducible assessment of amyloid burden. Quantification can enhance reader confidence, improve agreement among different readers, and assist in tracking changes in amyloid deposition over time [4]. The importance of quantification is increasingly acknowledged with the advent of Aβ-targeting drugs, such as lecanemab [5].

However, SUVr values cannot be directly compared when different radiotracers, regions of interest (ROIs), or image-processing algorithms are used [6]. To standardize quantification across tracers and institutions, the Centiloid (CL) scale was introduced in 2015 [7]. CL is intended to transform global cortical 50–70 min C-11 Pittsburgh Compound B (PiB) PET SUVr data into a scale that starts at 0, indicating individuals who are relatively “high certainty” amyloid-negative, and extends to 100, signifying individuals with confirmed amyloid accumulation in the cortex. This allows images from different amyloid PET tracers to be converted into a common metric, facilitating comparison and interpretation across studies. However, the original methodology necessitates technical proficiency and manual parameter adjustment, thereby restricting its routine application in clinical settings. For successful clinical integration, it is imperative to employ automated, user-friendly, and regulatory-compliant solutions.

Several commercial software platforms now provide automated or semi-automated CL quantification. MIM (v7.3.7, MIM Software Inc., Cleveland, OH, USA), with the MIMneuro^®^ package, provides semi-automated CL quantification from PET images, without the need for MRI scans. MIM has received regulatory approval from both the U.S. Food and Drug Administration (FDA) and the Korean Ministry of Food and Drug Safety (MFDS). MIM automatically aligns PET images to a standardized template. Visual inspection and adjustment of the alignment by the user are required before CL is calculated.

BTXBrain (v1.1.2, Brightonix Imaging Inc., Seoul, Korea), provides fully automated CL quantification from PET images, without the need for MRI scans. BTXBrain automatically transforms the PET images to a standardized template with an AI-based algorithm, and calculates CL values without user inspection [8]. BTXBrain is approved by the MFDS and is currently preparing for FDA approval.

SCALE PET 2.0 (v2.0.1, Neurophet Inc., Seoul, Republic of Korea) provides fully automated CL quantification using PET images. It is notable that SCALE PET requires 3D T1-weighted MRI images with 1 mm slice thickness for CL quantification. Patient MR images are automatically segmented to obtain cortical ROIs in individual space [9]. Then, PET images are co-registered to the MRI images to calculate the CL value without user inspection. SCALE PET 2.0 has been approved by the MFDS and is currently preparing for FDA approval.

While all three solutions claim good agreement with the CL values obtained from the original Centiloid method, when tested on the GAAIN (Global Alzheimer’s Association Interactive Network) database (http://www.gaain.org/centiloid-project, accessed on 13 Jan 2025), peer-reviewed validation studies remain limited.

The consistency of CL values from real-world clinical settings has not been sufficiently evaluated. Therefore, our study aimed to examine the agreement between CL values calculated by each software and determine whether the values from each software can be used interchangeably.

## 2. Materials and Methods

### 2.1. Patient Selection

**Figure 1:**
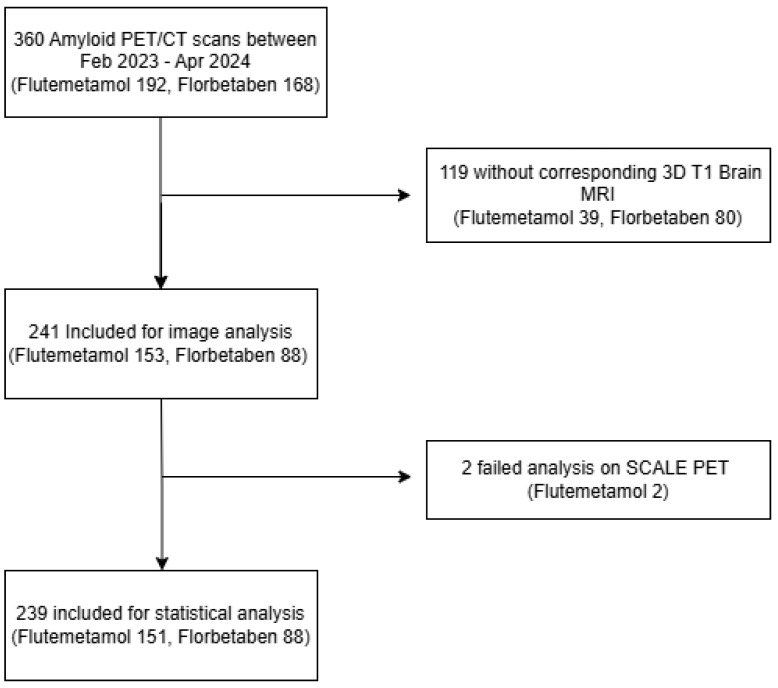
Flowchart of patient inclusion for the study.

This retrospective study was approved by the Institutional Review Board of our institution, and the requirement for informed consent was waived due to the retrospective nature of the study. This retrospective study included patients who underwent amyloid PET/CT between February 2023 and April 2024 at our institution. A total of 360 scans were initially identified, including 192 using F-18 flutemetamol (FMM) and 168 using F-18 florbetaben (FBB). Scans were excluded if a three-dimensional (3D) T1-weighted brain MRI was not available within 3 months of the PET/CT (FMM: *n* = 39; FBB: *n* = 80). Additionally, two FMM scans were excluded due to analysis failure using the Neurophet SCALE PET (v2.0.1, Neurophet Inc., Seoul, Republic of Korea) software. The final study cohort consisted of 239 patients (FMM: *n* = 151; FBB: *n* = 88). The flowchart is shown in Figure 1.

### 2.2. Imaging Protocol

The PET/CT scans were acquired with the Biograph Vision 600 system (Siemens Healthcare, Erlangen, Germany). Patients were intravenously administered either 185 MBq of FMM or 300 MBq of FBB. A 20 min PET scan was acquired 90 min after injection. Images were reconstructed using standard clinical protocols with iterative reconstruction and time-of-flight (TOF) correction. Partial volume correction was not applied.

For analysis using SCALE PET, all participants underwent 3D T1 MRI scans with 1 mm slice thickness, on either the Philips Achieva 1.5T, Philips Ingenio 3T, or Siemens Verio 3T scanner.

### 2.3. Imaging Analysis

All images were analyzed with the three commercially available software platforms: BTXBrain (v1.1.2, Brightonix Imaging Inc., Seoul, Republic of Korea), MIM (v7.3.7, MIM Software Inc., Cleveland, OH, USA) with MIMNeuro package, and SCALE PET. Each scan was processed independently using the three software platforms according to the respective manufacturer’s guidelines.

For CL quantification in BTXBrain, SUVRs were first calculated using the standard Centiloid cortical VOI and the whole cerebellum reference region by Klunk et al. [7]. The conversion to the CL scale is a two-step, platform-specific process. Initially, the SUVRs are converted using the standard public linear equations for FBB [10] and FMM [11]. Subsequently, BTXBrain performs an additional calibration by validating these initial CL values against the reference GAAIN dataset. A final, rescaled linear equation is then applied to ensure a one-to-one correspondence (i.e., a linear regression slope of 1.0 and y-intercept of 0.0) between the BTXBrain CL output and the reference standard.

Visual grading of amyloid PET images was conducted based on previous clinical reports documented at the time of image acquisition. These interpretations were provided by one of two board-certified nuclear medicine physicians with extensive experience. The scans were classified as either positive or negative for amyloid deposition using a binary classification system. In a limited number of cases where the findings were borderline, the reports were presented as ‘equivocal positive’ or ‘equivocal negative’, indicating the uncertainty in the visual interpretation.

### 2.4. Statistical Analysis

All statistical analyses were performed using R (v 4.3.3) and Python (v3.13). Linear regression was used to assess the correlation between CL values obtained from each pair of software platforms. Subsequently, Passing–Bablok regression analysis was performed for concordance. Statistically significant proportional error was present if the 95% confidence intervals (CI) of the slope from the regression equation did not include 1.0. Significant constant bias was present if the 95% CI for the y-intercept did not include 0.0.

Bland–Altman analysis was conducted to evaluate systematic bias and the limits of agreement between methods. Mean Centiloid values across the three software platforms were compared using repeated measures ANOVA followed by post hoc paired t-tests with Bonferroni correction. Subgroup analysis was performed for each radiotracer.

Centiloid values from each software were compared with a t-test according to visual interpretation status (visual-positive vs. visual-negative). A *p*-value of less than 0.05 was considered statistically significant.

## 3. Results

### 3.1. Demographics

Patient demographics are described in Table 1. A total of 239 patients were included in the study (99 males and 140 females). The median age was 76 years (range, 45–89 years). When stratified by tracer type, the FMM group included 151 patients (67 males and 84 females) with a median age of 76 years (range, 45–89), and the FBB group included 88 patients (32 males and 56 females) with a median age of 77 years (range, 54–89). Median time interval between PET and MR scan was 10 days. 

### 3.2. Image Analysis

Upon image analysis, two cases (both FMM) failed Centiloid analysis using SCALE PET due to registration failure. As SCALE PET is a fully automated solution, there is no method for manual adjustment once registration fails, and therefore these cases were excluded from the analysis. Upon visual inspection, both cases exhibited no remarkable anatomic abnormality on either PET or brain MRI. CL values were successfully obtained using both BTXBrain and MIM but were not included in the statistical analysis.

**Figure 2:**
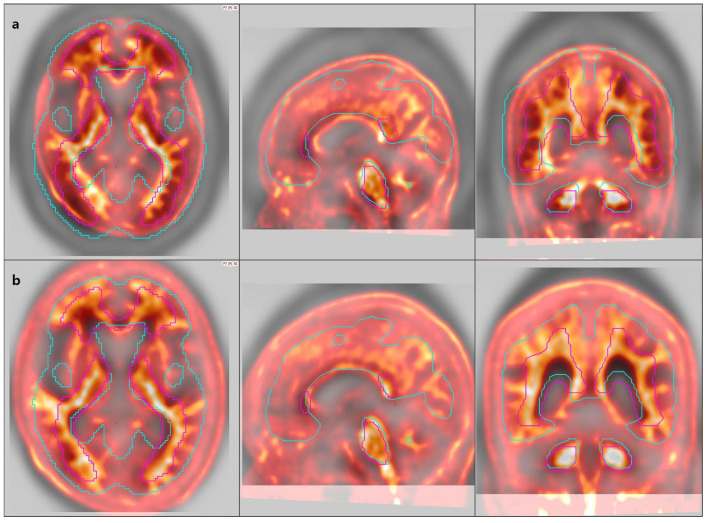
Example of PET-to-template misregistration in MIM analysis due to scalp uptake. The colored magenta and light blue represent the medullary and cortical VOIs, respectively. (**a**) Intense scalp uptake caused incorrect alignment, where the scalp was mistakenly registered as cortical brain tissue. (**b**) Image was manually adjusted.

**Figure 3:**
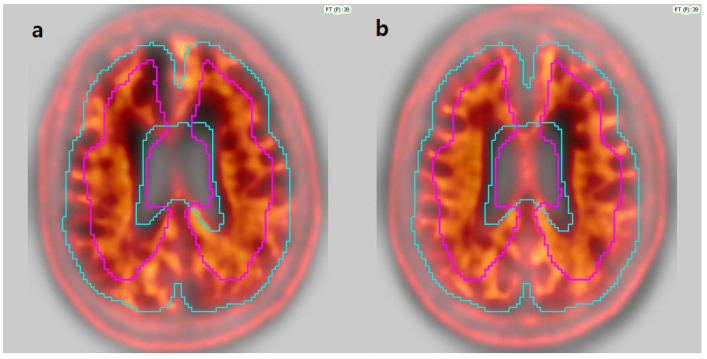
Example of PET-to-template misregistration in MIM due to incorrect image orientation. (**a**) The left panel shows the initial misaligned image caused by rotation. (**b**) Right panel shows the correctly aligned image after manual adjustment.

In addition, for the MIM software, manual registration correction was required in nine cases due to suboptimal alignment between PET and MRI images. Eight cases (7 FMM, 1 FBB) involved patients with prominent scalp uptake, which led to misregistration, where the scalp was mistakenly aligned with the cortical surface (Figure 2). One case required manual rotation due to incorrect image orientation (Figure 3). These findings indicate that while MIM allows user-guided registration correction, occasional manual intervention may be necessary to ensure accurate analysis.

### 3.3. Agreement Among Quantification Software

**Figure 4:**
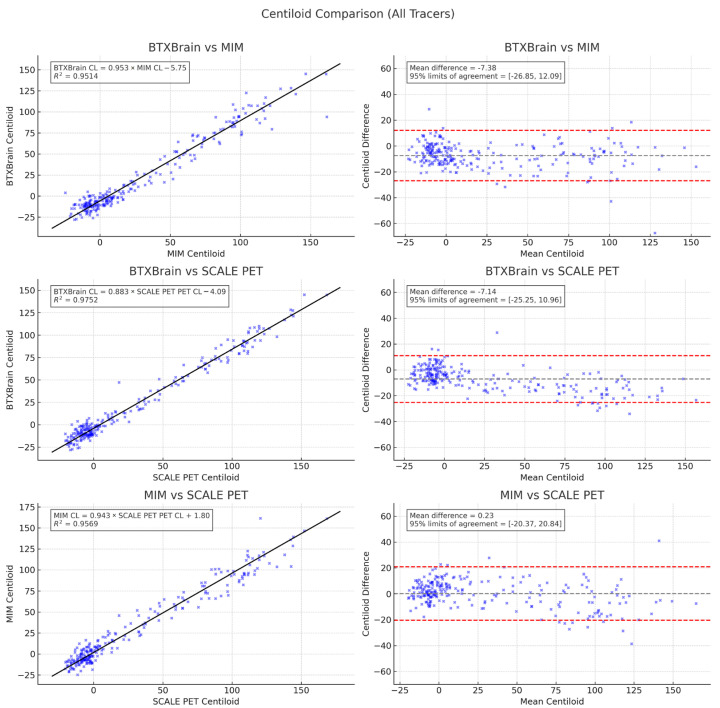
Scatter and Bland–Altman plots comparing Centiloid values from BTXBrain, MIM, and SCALE PET. Each data points are shown as blue x marking. The black lines in the Scatter plots represents the Passing–Bablok regression. Dotted red lines in the Bland–Altman plots represent 95% limits of agreement.

The correlation between Centiloid values derived from the three quantification programs (BTXBrain, MIM, and SCALE PET) is shown in Figure 4. A strong correlation was observed between BTXBrain vs. MIM (R^2^ = 0.9514), BTXBrain vs. SCALE PET (R^2^ = 0.9752), and MIM vs. SCALE PET (R^2^ = 0.9569). Passing–Bablok regression analysis performed for each pair of methods (BTXBrain vs. MIM, BTXBrain vs. SCALE PET, and MIM vs. SCALE PET) revealed the following regression equations:

BTXBrain CL = 0.953 (0.922 to 0.982) × MIM CL − 5.75 (−6.41 to −4.89)

BTXBrain CL = 0.883 (0.863 to 0.904) × SCALE PET CL − 4.09 (−4.53 to −3.68)

MIM CL = 0.943 (0.913 to 0.975) × SCALE PET CL + 1.80 (1.09 to 2.30)

The slope shows a significant proportional bias, where the CL values from BTXBrain were lower than those from MIM and SCALE PET as the CL value increased. The CL values from MIM were lower than those from SCALE PET, and higher than those from BTXBrain as the CL value increased. Bland–Altman plots visualize the proportional bias, especially between BTXBrain and SCALE PET.

**Figure 5:**
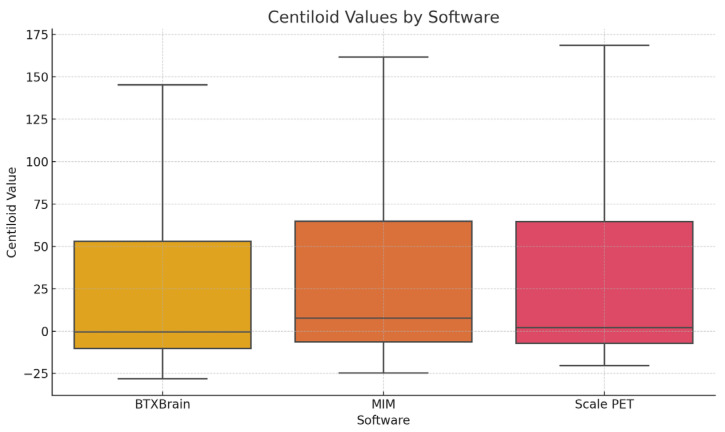
Box plots showing the distribution of Centiloid values from three software platforms.

Mean CL values were 21.89 ± 43.09, 29.26 ± 44.95, and 29.03 ± 48.82 for BTXBrain, MIM, and SCALE PET, respectively (Figure 5). Bonferroni-corrected pairwise comparisons show significant differences between BTXBrain vs. MIM (*p* < 0.001) and BTXBrain vs. SCALE PET (*p* < 0.001).

### 3.4. Subgroup Analysis by Radiotracer

**Figure 6:**
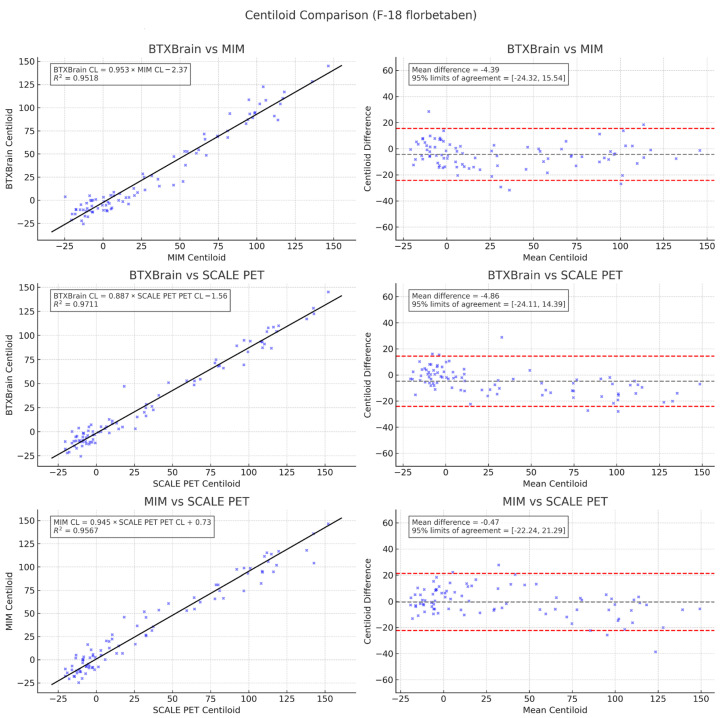
Scatter and Bland–Altman plots comparing Centiloid values derived from three different quantification programs in the florbetaben subgroup (*n* = 88).

**Figure 7:**
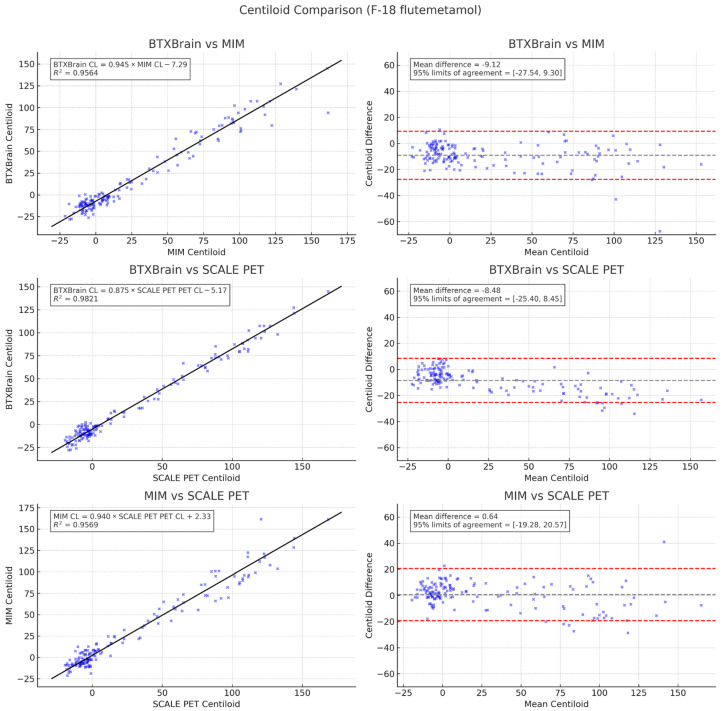
Scatter and Bland–Altman plots comparing Centiloid values among the three programs in the flutemetamol subgroup (*n* = 151).

Subgroup analysis by radiotracer, shown in Figure 6 and Figure 7, yielded similar results. A significant correlation was again observed between BTXBrain vs. MIM (R^2^ = 0.9518, 0.9564), BTXBrain vs. SCALE PET (R^2^ = 0.9711, 0.9821), and MIM vs. SCALE PET (R^2^ = 0.9567, 0.9569) for FBB and FMM, respectively. The slope of the two methods again showed proportional bias, with CL values from BTXBrain lower than those from SCALE PET and MIM as the CL value increased. The CL values from MIM were lower than those from SCALE PET, and higher than those from BTXBrain as the CL value increased.

Passing–Bablok regression for each pair of methods (BTXBrain vs. MIM, BTXBrain vs. SCALE PET, and MIM vs. SCALE PET) in the FBB subgroup was as follows:

BTXBrain CL = 0.953 (0.904 to 1.002) × MIM CL − 2.37 (−2.09 to 2.07)

BTXBrain CL = 0.887 (0.855 to 0.923) × SCALE PET CL − 1.56 (−1.87 to −0.50)

MIM CL = 0.945 (0.900 to 0.995) × SCALE PET CL + 0.73 (−0.71 to 2.12)

Passing–Bablok regression for each pair of methods (BTXBrain vs. MIM, BTXBrain vs. SCALE PET, and MIM vs. SCALE PET) in the FMM subgroup was as follows:

BTXBrain CL = 0.945 (0.905 to 0.980) × MIM CL − 7.29 (−8.38 to −6.59)

BTXBrain CL = 0.875 (0.850 to 0.898) × SCALE PET CL − 5.17 (−5.92 to −4.75)

MIM CL = 0.940 (0.899 to 0.988) × SCALE PET CL + 2.33 (1.60 to 2.72)

### 3.5. Centiloid Value Distribution According to Visual Assessment

**Figure 8:**
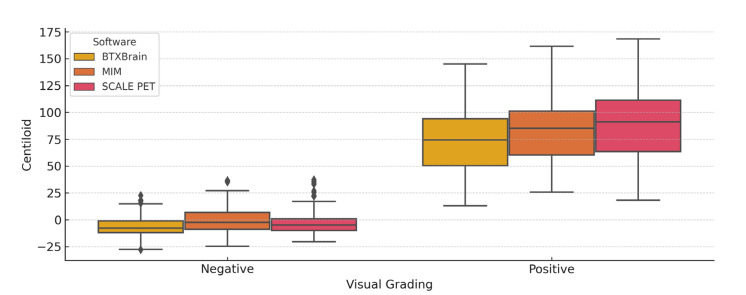
Box plots showing the distribution of Centiloid values from three software platforms according to visual interpretation results. The diamond symbols represent outliers beyond 2 standard deviation from mean.

The distribution of Centiloid values from each program by visual assessment was described in Figure 8 and Table 2. In all programs, the CL values in visually positive scans (*n* = 84) were clearly higher than those from visually negative (*n* = 155) scans. Among visually positive scans, CL values from BTXBrain were lower than those from SCALE PET and MIM, consistent with previously shown proportional bias.

Bonferroni-corrected pairwise comparisons in visually negative scans showed significant differences between all three programs (*p* < 0.001). Bonferroni-corrected pairwise comparisons in visually positive scans showed significant differences between all three programs (*p* < 0.001, *p* < 0.001, and *p* = 0.007 for BTXBrain vs. MIM, BTXBrain vs. SCALE PET, and MIM vs. SCALE PET, respectively).

### 3.6. Review of Individual Discordant Cases

**Figure 9:**
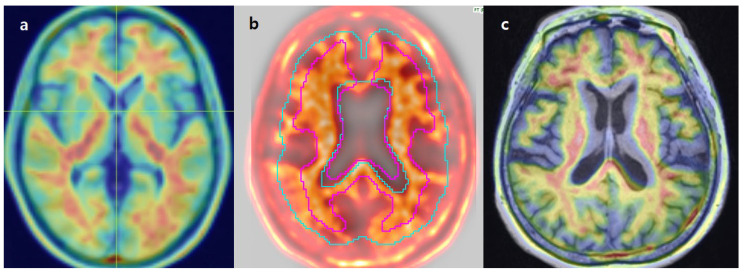
Registration of PET images on BTXBrain (**a**), MIM (**b**), and SCALE PET (**c**). In SCALE PET, PET images were misaligned with the anatomical MR image, while BTXBrain and MIM demonstrated proper alignment.

In case 1 (Figure 9), both the initial and repeated visual assessments were positive due to increased uptake in the posterior cingulate and lateral temporal cortex. The CL values from BTXBrain, MIM, and SCALE PET were 47, 46, and 18, respectively. While the exact cutoff for the interpretation of CL values is not established [12], CL values from BTXBrain and MIM would favor a positive scan, whereas the CL value from SCALE PET could be considered equivocal. Review of images in SCALE PET has shown mis-alignment between PET and MR images. As SCALE PET draws target ROIs over individual MR images, cortical uptake may result in underestimation of SUVr and CL values. The images were properly aligned in MIM and BTXBrain.

**Figure 10:**
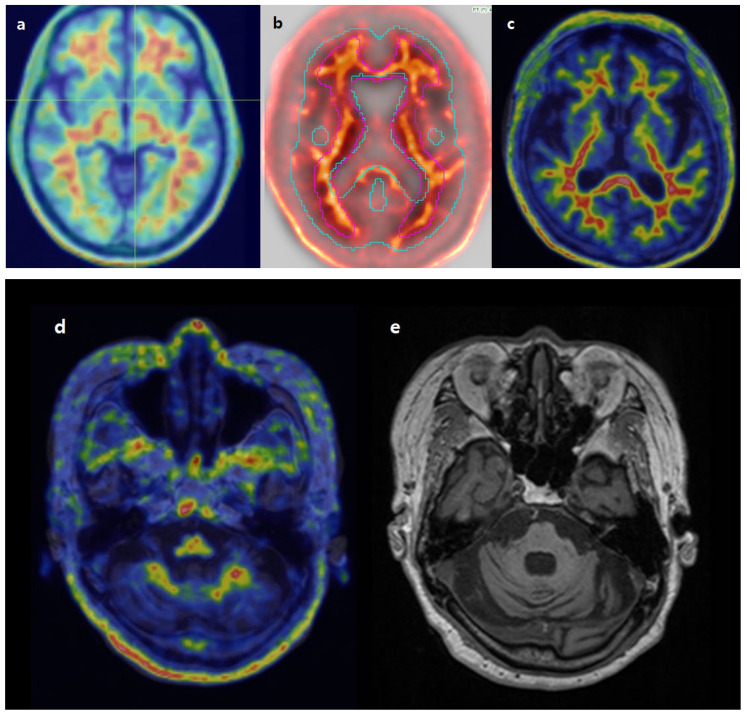
Registration of PET images on BTXBrain (**a**), MIM (**b**), and SCALE PET (**c**). BTXBrain, MIM, and SCALE PET all show acceptable alignment. Fused PET/MR (**d**) shows decreased cerebellar uptake. Patient MR (**e**) shows cerebellar atrophy.

In case 2 (Figure 10), both initial and repeated visual evaluations were clearly negative. The CL values obtained from BTXBrain, MIM, and SCALE PET were 23, 36, and 37, respectively. Image alignment was acceptable in all three software programs. Despite preservation of gray matter-to-white matter contrast, there was a reduction in overall uptake in the cerebellum, attributable to cerebellar atrophy.

As all three software platforms use the whole cerebellum as the reference region, this decreased cerebellar uptake likely led to a systematic overestimation of Centiloid values across all methods.

## 4. Discussion

In this study, we evaluated the consistency of Centiloid values calculated using three commercially available software programs: BTXBrain (v1.1.2), MIM (v7.3.7), and SCALE PET (v2.0.1). Our results demonstrated a high level of agreement among the calculated CL values, with strong correlation coefficients. However, systematic bias was noted, where CL from SCALE PET was notably higher as the CL value increased, followed by CL from MIM, and CL from BTXBrain.

Centiloid scaling is designed as a standardized quantitative approach for interpreting amyloid PET/CT images, enabling consistent assessment across different tracers, scanners, and institutions [7]. It facilitates objective diagnosis and longitudinal monitoring of β-amyloid burden, supporting both clinical decision-making and research comparability.

The clinical significance of CL quantification is increasingly recognized, particularly as anti-amyloid therapies are introduced [5]. Some recent trials developing novel therapeutic agents for AD have incorporated CL values for patient selection, dose determination, and treatment cessation [13,14]. However, the original Centiloid processing pipeline based on statistical parametric mapping (SPM) is time-consuming and technically demanding. As healthcare professionals, including nuclear medicine physicians, are under a consistently increasing workload [15], a time-consuming process is impractical for routine clinical use. This limitation has prompted the development of several automated or semi-automated software solutions aimed at streamlining CL calculations in real-world settings. Artificial intelligence (AI)-based auto-quantification has also been developed for CL calculation [16].

Recently, several commercially available software solutions have been developed for automatic and semi-automatic CL calculations. They are designed to produce structured reports without the need for extensive labor. However, the consistency and reliability of the CL values produced by each software program have not been extensively evaluated.

In many cases, CL calculation functionality has only recently been integrated, and regulatory approval processes are still ongoing. Consequently, the availability of each program varies by country. For example, MIM has obtained both MFDS (Korea) and FDA (U.S.) clearances for CL calculations. In contrast, the most recent Centiloid-enabled versions of BTXBrain and SCALE PET have been cleared by the MFDS but are not yet cleared by the FDA. On the other hand, some software, such as Syngo.PET (Siemens), which was not included in the present study, has received FDA clearance but has not yet been cleared by the MFDS for their update, including CL calculation functionality. In our study, we evaluated BTXBrain, MIM, and SCALE PET, which were available at our institution.

The correlation of CL values across the software was very high. This suggests that the analysis pipeline of all three software programs is generally robust. This is in accordance with previously published data, where SUVr derived from each software was well correlated with research tools [8,9,17,18]. However, there was a significant proportional bias, where SCALE PET tended to yield relatively higher CL values and BTXBrain tended to yield relatively lower CL values as CL increased. This trend was consistent across both FMM and FBB subgroups, suggesting that the observed bias is not tracer-dependent. This indicates that the cause of the discrepancy is likely the Centiloid conversion equation within each software package not aligning well with our study dataset. This is particularly intriguing, as each software vendor states (with unpublished data) that their software is well calibrated against the original Centiloid method on the GAAIN database, with high agreement and without significant systematic bias. This may indicate that each software reacts differently to the differences between the GAAIN database and our study data.

It should be noted that despite its significant strength and wide adoption in the research field, the Centiloid has some remaining challenges. One issue is verifying the inconsistency between different scanners, particularly digital detectors. The key component in the development of the Centiloid method and its calibration is the publicly available GAAIN database, which mainly consists of images on conventional PET/CT scanners with comparable resolution. The assumption within the Centiloid process is that any subject would yield the same SUVr values if scanned at two different sites or scanners several days apart [7], but this assumption was not validated, and the authors acknowledge it as their critical limitation. Gillman et al. evaluated the effect of different PET scanners on the CL value calculated using the original Centiloid method [19]. Their study showed a significant proportional bias, where CL from Siemens Vision was notably higher compared with those from Siemens mCT or Philips Gemini as the CL value increased. Their study also found that the amount of bias was different with different CL calculation programs. Their study suggests that scanner differences, especially with recent digital scanners, can produce bias in CL calculation and that different programs may react differently.

With recent advances in PET technology, various scanners with different architectures have been introduced. These include digital PET/CT systems with silicon photomultipliers [20,21], dedicated brain PET scanners [22], and large axial field-of-view scanners [23]. It is possible that these scanners exhibit different imaging characteristics that may affect CL calculation. Further research on the potential scanner effect on different CL calculation software may improve the consistency and reproducibility of the Centiloid in the clinic. Before these effects are fully addressed, it may be reasonable to use the same scanner and software for CL calculation within longitudinal monitoring and studies, such as treatment response assessment, when feasible.

The differences between the target and reference ROIs are also discussed. MIM utilizes the amyloid processing approach described by Navitsy et al. [24] to determine the global cortical SUVr value, averaging six VOIs (medial orbital frontal, lateral temporal, superior parietal, anterior cingulate, posterior cingulate, and precuneus) relative to the whole cerebellum. BTXBrain employs an AI-based algorithm to automatically transform PET images into a standardized template without the need for patient MR images. The original cortical target region defined by Klunk et al. [7] was used to calculate the SUVr with reference to the whole cerebellum. SCALE PET requires patient MRI images acquired with 3D T1-weighted volumetric MRI for Centiloid calculation. These MRI images underwent automatic segmentation to determine the target ROI. Following the co-registration of patient PET and MRI images, the target ROI was employed to calculate the SUVr with reference to the entire cerebellum.

Shekari et al. [25] compared 32 CL pipelines with various target and reference ROIs based on SPM12. Their study showed that the selection of the reference region had the highest impact on CL values and that the whole cerebellum reference and whole cerebellum plus brainstem reference yielded robust results. All the software tested in our study used the whole cerebellum as the reference region. The high level of correlation seen in our data also shows the robustness between the software programs. In their study, a subject-based cortical target ROI rendered CL values more sensitive to the harmonization status, which suggests that SCALE PET’s pipeline may be more sensitive to the resolution of the PET scanner.

The Centiloid values from all three programs were generally in accordance with visual assessment. Studies identified as negative had low CL values, and only three visually negative cases reported CL values over 30. Two of the three cases were reported as “equivocal negative”, indicating the difficulty of qualifying the amyloid PET image within a binary system of positive or negative [26]. One truly discrepant case was case 2, where the image was confidently reported as negative. Both nuclear medicine physicians confidently classified the image as negative, even when retrospectively reviewing with knowledge of the CL values. After detailed inspection of PET and MR images for the cause of this discrepancy, we concluded that the Centiloid value appeared to have been overestimated, likely due to cerebellar atrophy, which may have influenced the reference region employed in the SUVR calculation. This instance underscores a potential limitation of CL quantification, particularly in patients exhibiting structural brain changes. In addition, as seen in case 1, inaccurate image registration can also result in erroneous Centiloid values. Consequently, when interpreting Centiloid values, it is essential to integrate quantitative results within the broader clinical context. Visual assessment by experienced readers, along with verification of registration accuracy and consideration of structural abnormalities on MRI or CT, should complement automated measurements to prevent misclassification.

The clinical applicability of each software study should also be considered. The clinical usefulness of the Centiloid would decrease if CL values were unavailable in many patients. Notably, MIM exhibited the highest rate of automatic registration failure, occurring in 9 out of 241 cases (3.7%). However, the user could manually modify the registration, which allowed for CL calculations in all instances. SCALE PET showed two instances (0.8%) of CL calculation failure and one instance (0.4%) of visible misregistration. This is similar to the reported registration failure rate (1.2%) [9]. Although the incidence was lower compared to MIM, the lack of manual adjustment capability limited the availability of CL calculation in those cases. In addition, the need for 3D MR images also limits clinical availability, as shown in our dataset where almost one-third—119 out of 360 (33.0%)—were excluded from analysis due to the absence of appropriate MR images. Notably, there were no instances of calculation failure or notable misregistration of images with BTXBrain in our dataset.

A crucial consideration for BTXBrain is the application of level 2 calibration for the Centiloid conversion formula. By default, BTXBrain uses pre-established standard public linear equations for FBB [10] and FMM [11], which were established using the original Centiloid method. However, even when the same target VOI and equation are used, the differences between image processing pipelines may result in different SUVr and, consequently, Centiloid values. Battle et al. compared different image processing pipelines and developed alternate equations for different pipelines [27]. The Centiloid project provides an image dataset for level 2 calibration on the GAAIN database and recommends that new image analysis methods undergo this process [7]. At our request, BTXBrain provided a new slope calibrated against the GAAIN reference dataset. Without level 2 calibration, the software’s default equation clearly underestimates CL values compared to the other platforms (Supplementary Materials, Figures S1–S3). We recommend that other BTXBrain users adopt this GAAIN-calibrated slope to improve inter-software consistency and ensure the comparability of results.

Currently, there is very limited literature on the consistency of Centiloid values calculated with various programs. Shang et al. assessed the agreement among three CL values from three research tools and showed high correlation between the methods with little systematic bias [28]. To our knowledge, our study is the first to compare the consistency of Centiloid values from commercially available software solutions.

Our study had several limitations. First, it was a retrospective analysis that exclusively included patients who underwent amyloid PET/CT scans at a single institution. Notably, all participants were of a single ethnicity, which restricts the generalizability of our findings to a broader clinical context. Second, our study was conducted using a single PET/CT scanner. This restricts the generalizability of our findings. Future multicenter studies using a variety of PET/CT systems would be valuable for assessing the consistency of Centiloid quantification across different imaging environments. Furthermore, our study did not account for potential confounding factors such as cognitive status, comorbidities, or medication use, which could influence amyloid deposition and PET/CT results.

A significant limitation of our study is the absence of pathological verification and direct comparison with the original SPM-based Centiloid pipeline. Although our study identified a proportional bias among the CL values generated by the three software programs, it remains unclear which software provides the most accurate results due to the lack of a true reference standard. Although postmortem validation offers the most precise verification of amyloid burden, it is impractical for routine clinical practice, and data incorporating postmortem analysis are exceedingly limited. Although the original Centiloid method pipeline was validated using postmortem data and may serve as an alternative to postmortem validation, the absence of digital PET/CT data within the GAAIN database, coupled with the reported bias of the original Centiloid pipeline from digital PET/CT scanners [19], suggests that even comparison with the original Centiloid pipeline may be inadequate as a true reference standard. To our knowledge, no postmortem validation study has been conducted for digital PET/CT scanners. Further evaluation of the various scanner effects and CL calculation methods is warranted.

## 5. Conclusions

The three commercial software programs—BTXBrain, MIM, and SCALE PET—demonstrated high consistency in Centiloid quantification. However, there was a notable systematic bias. These findings support the robustness of automated pipelines while also underscoring the need to consider their limitations and the contextual factors that may influence CL values. Before these effects are fully addressed, it may be reasonable to use the same scanner and software for CL calculation within longitudinal monitoring and studies, when feasible.

## Figures and Tables

**Table 1:** Patient demographics.

Group	*n* (Male/Female)	Age, Median (Range)
Total	239 (99/140)	76 (45–89)
Flutemetamol	151 (67/84)	76 (45–89)
Florbetaben	88 (32/56)	77 (54–89)

**Table 2:** Comparison of Centiloid values in visually negative and positive groups.

	Visually Negative	Visually Positive	*p*-Value
BTXBrain	−6.3 ± 9.9	73.9 ± 30.4	<0.001
MIM	−0.2 ± 12.1	83.6 ± 30.3	<0.001
SCALE PET	−3.0 ± 11.3	88.2 ± 33.8	<0.001

## Data Availability

The data presented in this study are available on request from the first author. The data are not publicly available due to privacy restrictions on clinical information.

## Supplementary Materials

The following supporting information can be downloaded at https://www.mdpi.com/article/10.3390/diagnostics15131599/s1, Figure S1: Scatter and Bland–Altman plots comparing Centiloid values from BTXbrain in default setting, MIM and SCALE PET; Figure S2: Scatter and Bland–Altman plots comparing Centiloid values from BTXbrain in default setting, MIM and SCALE PET, in the florbetaben subgroup (n = 88).; Figure S3: Scatter and Bland–Altman plots comparing Centiloid values from BTXbrain in default setting, MIM and SCALE PET, in the flutemetamol subgroup (*n* = 151).
