# Supplementary material for: Consistency Analysis of Centiloid Values Across Three Commercial Software Platforms for Amyloid PET Quantification
Source: Diagnostics (Basel). 2025 Jun 24;15(13):1599. doi: 10.3390/diagnostics15131599 (PMC12248461; doi:10.3390/diagnostics15131599)
Supplement: Supplementary file 1 [file diagnostics-15-01599-s001.zip › diagnostics-3668416-supplementary.pdf]

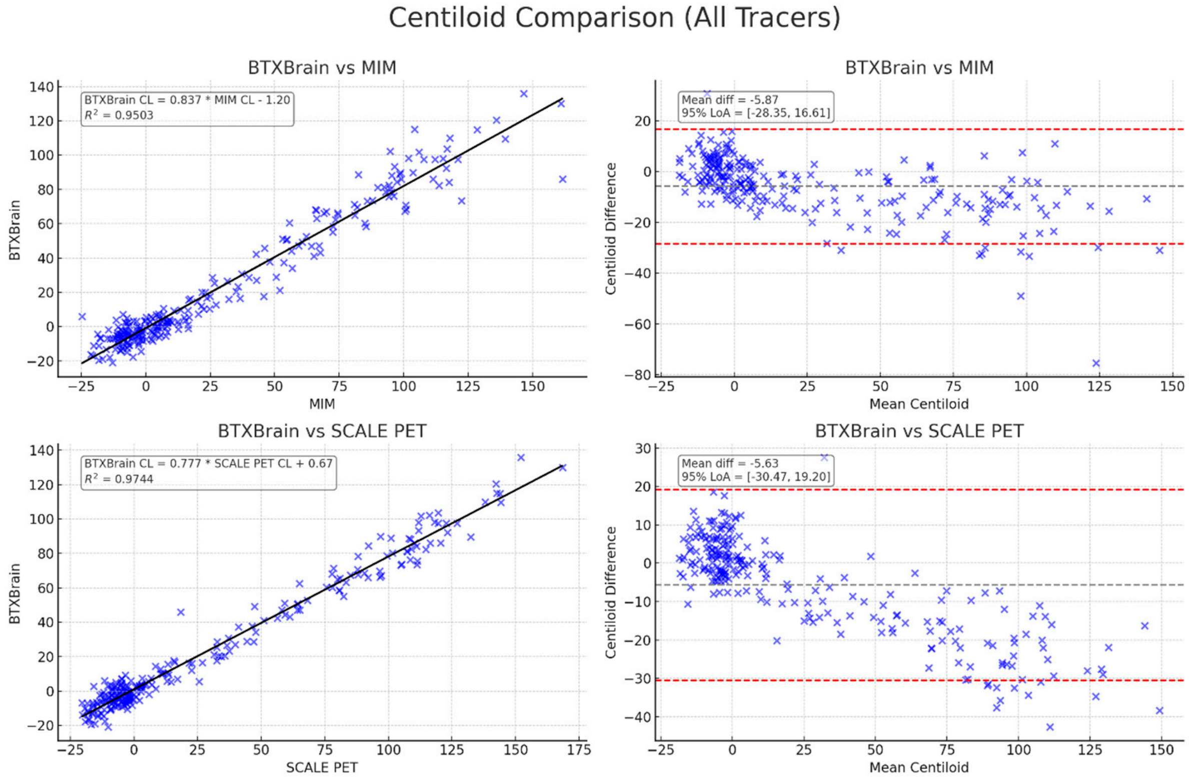

Figure S1. Scatter and Bland–Altman plots comparing Centiloid values from BTXbrain in default setting, MIM and SCALE PET.

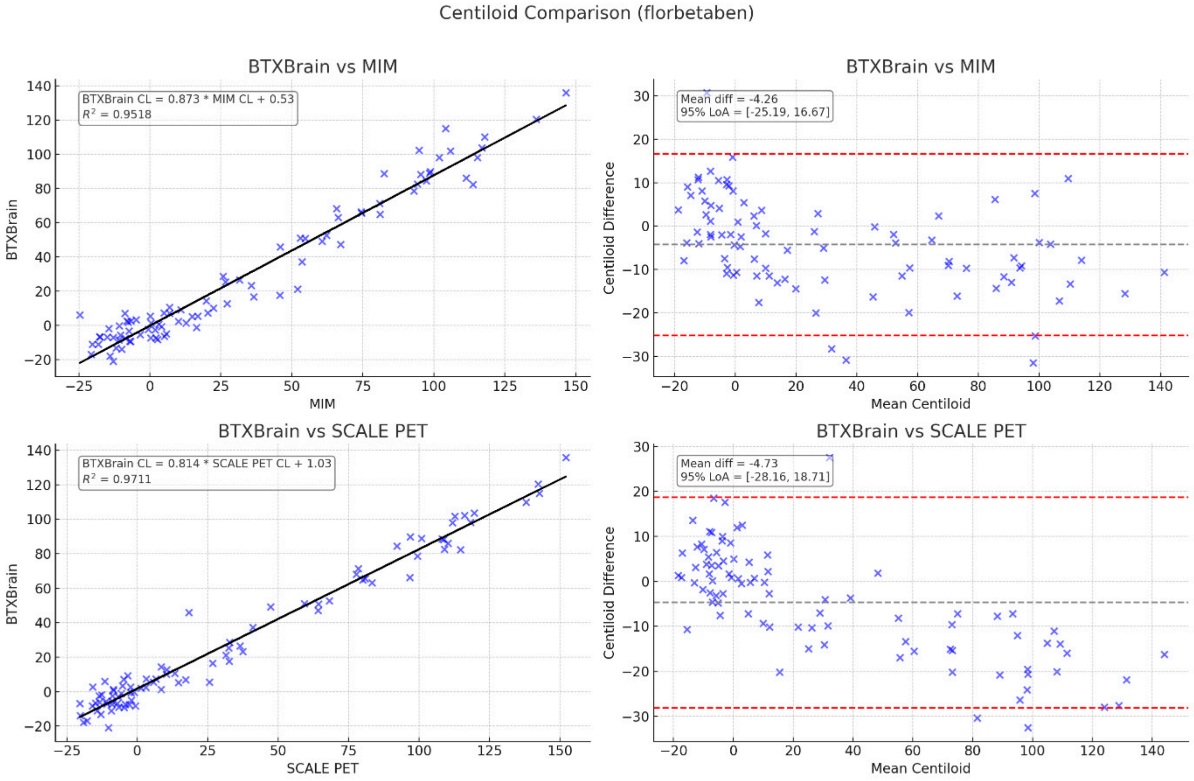

Figure S2. Scatter and Bland–Altman plots comparing Centiloid values from BTXbrain in default setting, MIM and SCALE PET, in the florbetaben subgroup (n = 88).

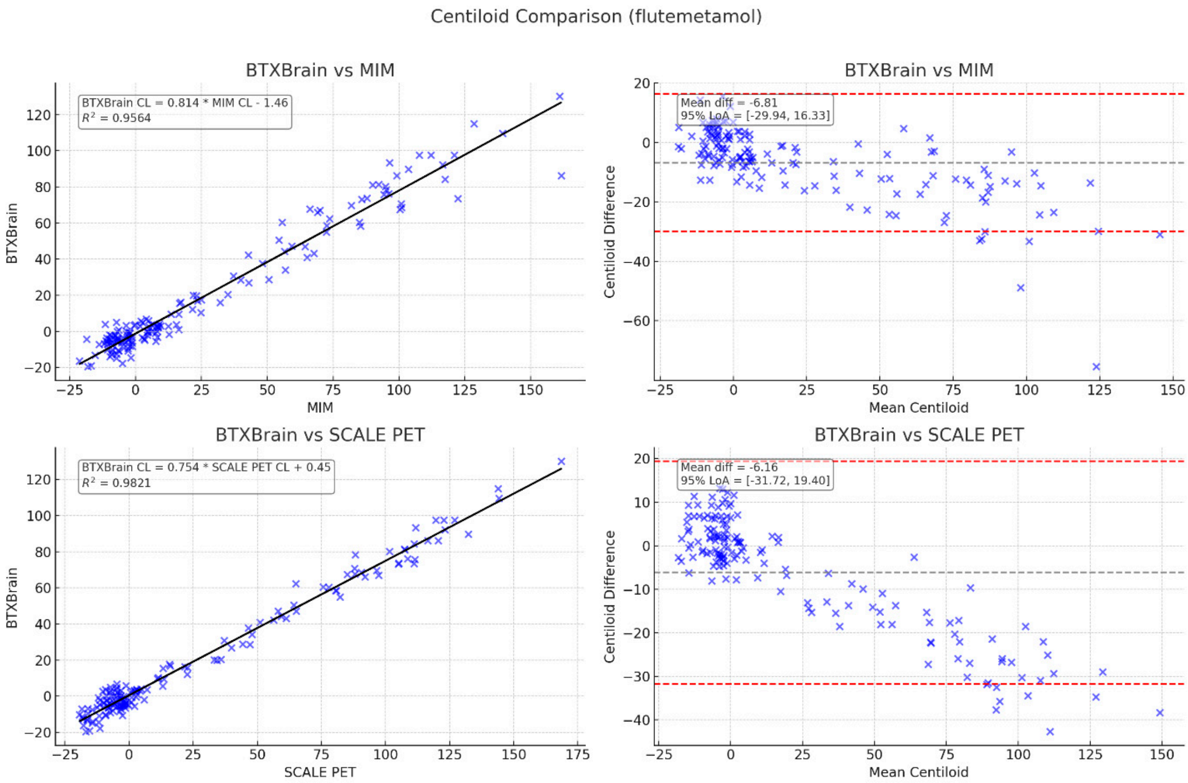

Figure S3. Scatter and Bland–Altman plots comparing Centiloid values from BTXbrain in default setting, MIM and SCALE PET, in the flutemetamol subgroup (n = 151).
